# Selection of reference genes for qRT-PCR examination of wild populations of Atlantic cod *Gadus morhua*

**DOI:** 10.1186/1756-0500-1-47

**Published:** 2008-07-16

**Authors:** Pål A Olsvik, Liv Søfteland, Kai K Lie

**Affiliations:** 1 National Institute of Nutrition and Seafood Research, Nordnesboder 2, N-5005 Bergen, Norway

## Abstract

**Background:**

Extensive sequencing efforts have been taking place for the Atlantic cod (*Gadus morhua*) in recent years, the number of ESTs in the Genbank has reached more than 140.000. Despite its importance in North Atlantic fisheries and potential use in aquaculture, relatively few gene expression examination exists for this species, and systematic evaluations of reference gene stability in quantitative real-time RT-PCR (qRT-PCR) studies are lacking.

**Results:**

The stability of 10 potential reference genes was examined in six tissues of Atlantic cod obtained from four populations, to determine the most suitable genes to be used in qRT-PCR analyses. Relative transcription levels of genes encoding β-actin (ACTB), elongation factor 1A (EF1A), actin-related protein-2 (ARP-2), glyceraldehyde-3P-dehydrogenase (GAPDH), ubiquitin (Ubi), acidic ribosomal protein (ARP), ribosomal protein S9 (S9), ribosomal protein L4 (RPL4), RPL22 and RPL37 were quantified in gills, brain, liver, head kidney, muscle and middle intestine in six juvenile fish from three wild populations and from farmed Atlantic cod. Reference gene stability was investigated using the *geNorm *and *NormFinder *tools. Based on calculations performed with the *geNorm*, which determines the most stable genes from a set of tested genes in a given cDNA sample, ARP, Ubi, S9 and RPL37 were among the most stable genes in all tissues. When the same calculations were done with *NormFinder*, the same genes plus RPL4 and EF1A were ranked as the preferable genes.

**Conclusion:**

Overall, this work suggests that the Ubi and ARP can be useful as reference genes in qRT-PCR examination of gene expression studying wild populations of Atlantic cod.

## Background

The specificity and ease-of-use of quantitative reverse transcription real-time PCR (qRT-PCR) has made this method the dominant technique for quantification of relative mRNA levels in cells. In qRT-PCR, the expression levels of the target genes of interest are normally estimated on the basis of endogenous controls, also called reference genes. The purpose of these controls is to remove or reduce differences due to biological and technical variability, i.e. differences in RNA quantity and quality. The ideal endogenous control should be expressed at a constant level at all stages of development or between treatment groups. It should also be expressed at roughly the same level as the mRNA under study. Hence, data normalization is a prerequisite of the qRT-PCR analytical process, and is essential for accurate comparison of mRNA measurements between different samples [1,2]. However, no golden standard exists for normalization of qRT-PCR data, and data normalization remains a real problem in qRT-PCR. Numerous studies have shown that no single universal gene has a constant expression level under all developmental or experimental conditions. The best choice of reference genes to be used as endogenous control varies, depending on the cells or tissues studied, and has to be validated in each experiment. Many different genes have therefore been selected for normalization of mRNA expression data [3-7]. If the selected reference genes fluctuate randomly between treatment groups, differences in expression between the genes of interest will be missed.

The Atlantic cod (*Gadus morhua*) has a wide distribution across the North Atlantic [8]. Several important cod stocks are of great economic and social importance. As a consequence, Canadian and Norwegian groups have sequenced more than 140.000 ESTs in this species. For the time being, there are no reports on suitable reference genes for qRT-PCR examinations of wild-caught Atlantic cod available. The aim of this work was therefore to evaluate the usefulness of 10 potential reference genes for qRT-PCR in the Atlantic cod. Our hypothesis was that reference genes are stable in tissues within and between populations of cod living in contaminated habitats. Genes encoding 5 commonly used housekeeping proteins plus 5 ribosomal proteins were selected for examination. To evaluate their usefulness as reference genes, RNA from six tissues (gill, brain, liver, head kidney, muscle and intestine) of six adult male cod from four populations were subjected to qRT-PCR analysis [see Additional file 1 for methods]. Two of the populations were sampled from heavily contaminated recipients, one control from an unpolluted fjord locality and one from an aquaculture facility. We used the *geNorm *and *NormFinder *tools to evaluate the individual stability of the reference genes, ranking the genes according to their usefulness as reference genes in qRT-PCR examinations.

## Results and Discussion

Even if alternatives exist it is becoming more evident today that in order to obtain trustworthy gene expression data using real-time RT-PCR it is necessary to normalize the data with internal control genes, based on their mRNA levels. Every examination therefore requires that one quantify one or even better two or more reference genes and evaluate their expression stability in order to normalize the expression data. In the current examination the stability of 10 potential reference genes (Table 1, Table 2) were screened in six tissues of Atlantic cod sampled from four different populations in Western Norway, two of them from locations contaminated with xenobiotics. This experiment represents the first attempt to characterize and evaluate potential reference genes in qRT-PCR studies of Atlantic cod. Fish from three different wild populations as well as farmed fish were studied. Sediments in Store Lungegårdsvann in Bergen town and in Sørfjorden close to Odda are contaminated with POPs and heavy metals that might affect fish inhabiting these locations. Biota from these locations are affected by the pollutants [9,10]. Farmed cod will of course have a steady supply of food, and will in general be in a better condition than wild cod. For future qRT-PCR examinations of wild cod, it is a prerequisite to have candidate reference genes that exhibit stable expression across different populations.

**Table 1:** Reference genes evaluated in the present study. Gene symbol, name and function are shown.

**Symbol**	**Gene name**	**Function**
ACTB	β-actin	Cytoskeletal structural protein
ARP-2	Actin-related protein-2	Cytoskeletal structural protein
EF1A	Elongation factor 1 alpha	Protein synthesis
Ubi	Ubiquitin	Protein degradation
GAPDH	Glyceraldehyde-3-phosphate dehydrogenase	Glycolytic protein
RPS9	Ribosomal protein S9	Member of ribosome proteins
ARP	Acidic ribosomal protein	Member of ribosome proteins
RPL4	Ribosomal protein L4	Member of ribosome proteins
RPL22	Ribosomal protein L22	Member of ribosome proteins
RPL37	Ribosomal protein L37	Member of ribosome proteins

**Table 2:** qRT-PCR assays used to evaluate potential reference genes in Atlantic cod. Gene short names, Genbank accession numbers, PCR primer sequences, amplicon sizes and PCR efficiencies (mean ± STDEV in six tissues) are shown.

**Gene**	**Accession no.**	**Forward primer (5' – 3')**	**Reverse primer (5' – 3')**	**Amplicon size (bp)**	**PCR eff.**
ACTB	EX739174	CACAGCCGAGCGTGAGATT	ACGAGCTAGAAGCGGTTTGC	95	2.03 ± 0.09
ARP-2	EX741634	TCTGCTCCGTGTGGAAGTTG	CGAGAAGATCCTCTGCCACAA	131	2.06 ± 0.04
EF1A	EX721840	CCCTGTGGAAGTGGCTGAAG	CATCCAAGGGTCCGTATCTCTT	93	2.02 ± 0.11
Ubi	EX735613	GGCCGCAAAGATGCAGAT	CTGGGCTCGACCTCAAGAGT	69	1.91 ± 0.09
GAPDH	EX725566	CCATGACAACTTTGGCATCGT	AGGGTCCGTCCACTGTCTTCT	83	2.16 ± 0.07
RPS9	EX726043	TCTTTGAAGGTAATGCTCTGTTGAGA	CGAGGATGTAATCCAACTTCATCTT	84	1.94 ± 0.08
ARP	EX741373	TGATCCTCCACGACGATGAG	CAGGGCCTTGGCGAAGA	113	1.95 ± 0.10
RPL4	EX725958	GGTGCCATACAGCTGATCCA	CCAGGCATCACACTGCAGAA	123	1.98 ± 0.12
RPL22	EX727868	GTTACCGGTCTTCCCGTTGA	AGAAGTCCAAAAAAGGAGCTTCCT	132	1.79 ± 0.08
RPL37	EX738140	CCGAGAAGCGCAAGAGAAAG	GGTGGTACCTTCCCGGAATC	134	1.87 ± 0.06

**Figure 1:**
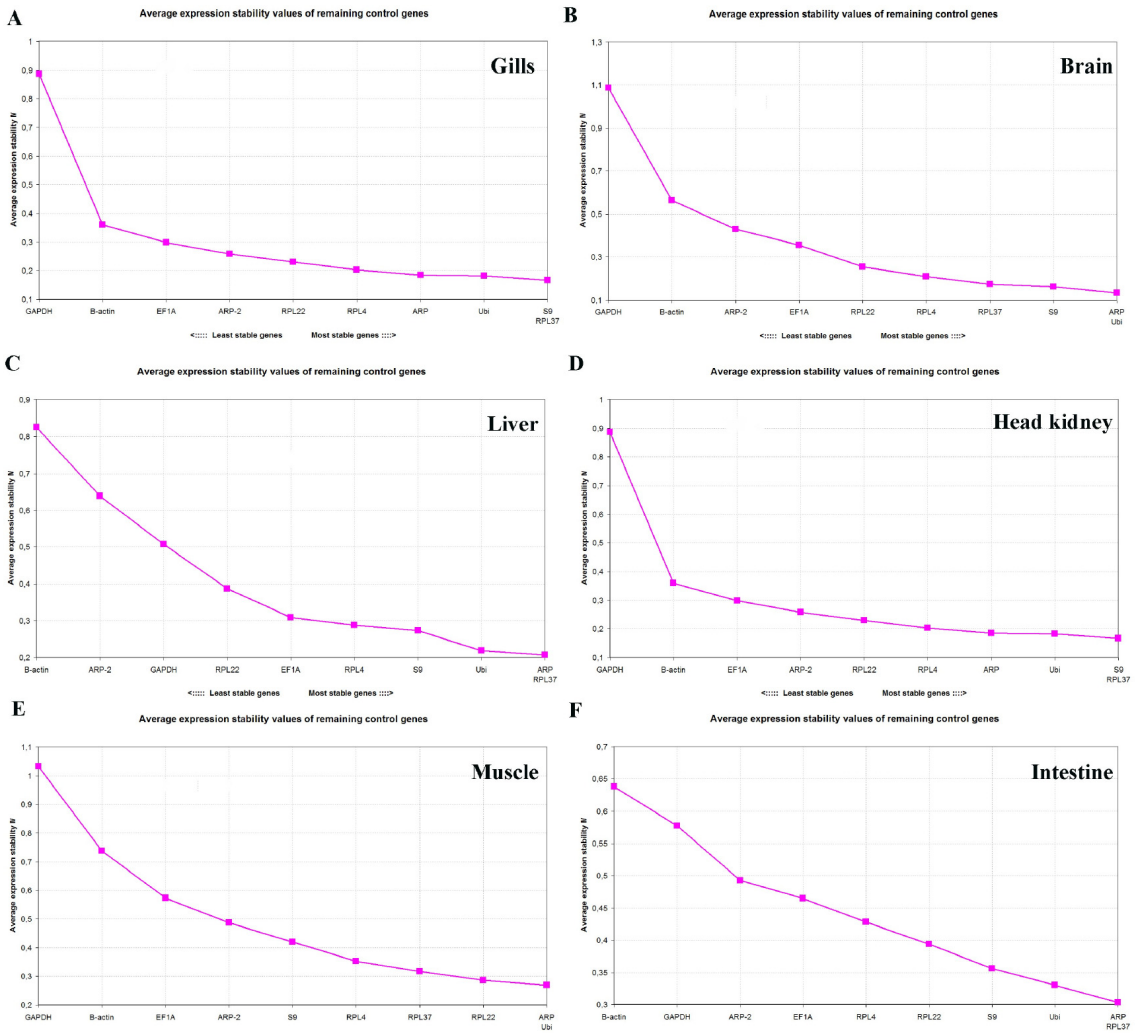
Ranking of the reference genes according to the *geNorm *software, all samples. A) Gills, B) Brain, C) Liver, D) Head kidney, E) Muscle and F) Intestine.

Ranking of the most stable reference genes in gills, brain, liver, head kidney, muscle and intestine analyzed by *geNorm *is presented in Fig. 1. The *geNorm *software ranks the selected reference genes according to the determined gene-stability measure (M-value), representing the average pair-wise variation of a particular gene with all other reference genes, from the most stable (lowest M values) to the least stable (highest M values) [5]. The best two genes are ranked without distinguishing between them. In gills, Ubi and RPL37 ranked best with S9 ranking number three (Fig. 1A). The two worst genes were GAPDH and ACTB. ARP and Ubi followed by S9 were the most stably expressed genes in brain tissue according to *geNorm*, whereas GAPDH and ACTB ranked worst (Fig. 1B). In liver ARP and RPL37 were ranked as the two best genes, with Ubi ranking number three (Fig. 1C). ACTB and ARP-2, two genes encoding structural proteins, were ranked as the most unsuitable reference genes in liver. Two ribosomal protein genes ranked best in head kidney tissue, S9 and RPL37, followed by Ubi, whereas GAPDH and ACTB again were classified as the most unstable genes (Fig. 1D). In muscle ARP and Ubi were the best genes, followed by RPL22 (Fig. 1E). ARP and Ubi were also ranked as the best genes in brain tissue. Again GAPDH and ACTB were estimated to be the least stable genes. ARP and RPL37 were the most stable reference genes in intestinal tissue, with Ubi ranking number third (Fig. 1F). Also in this tissue ACTB and GAPDH were the worst genes. Overall, according to the *geNorm *algorithm genes encoding ribosomal proteins plus Ubi should be used for normalization of qRT-PCR data in the six examined tissues of Atlantic cod.

**Figure 2:**
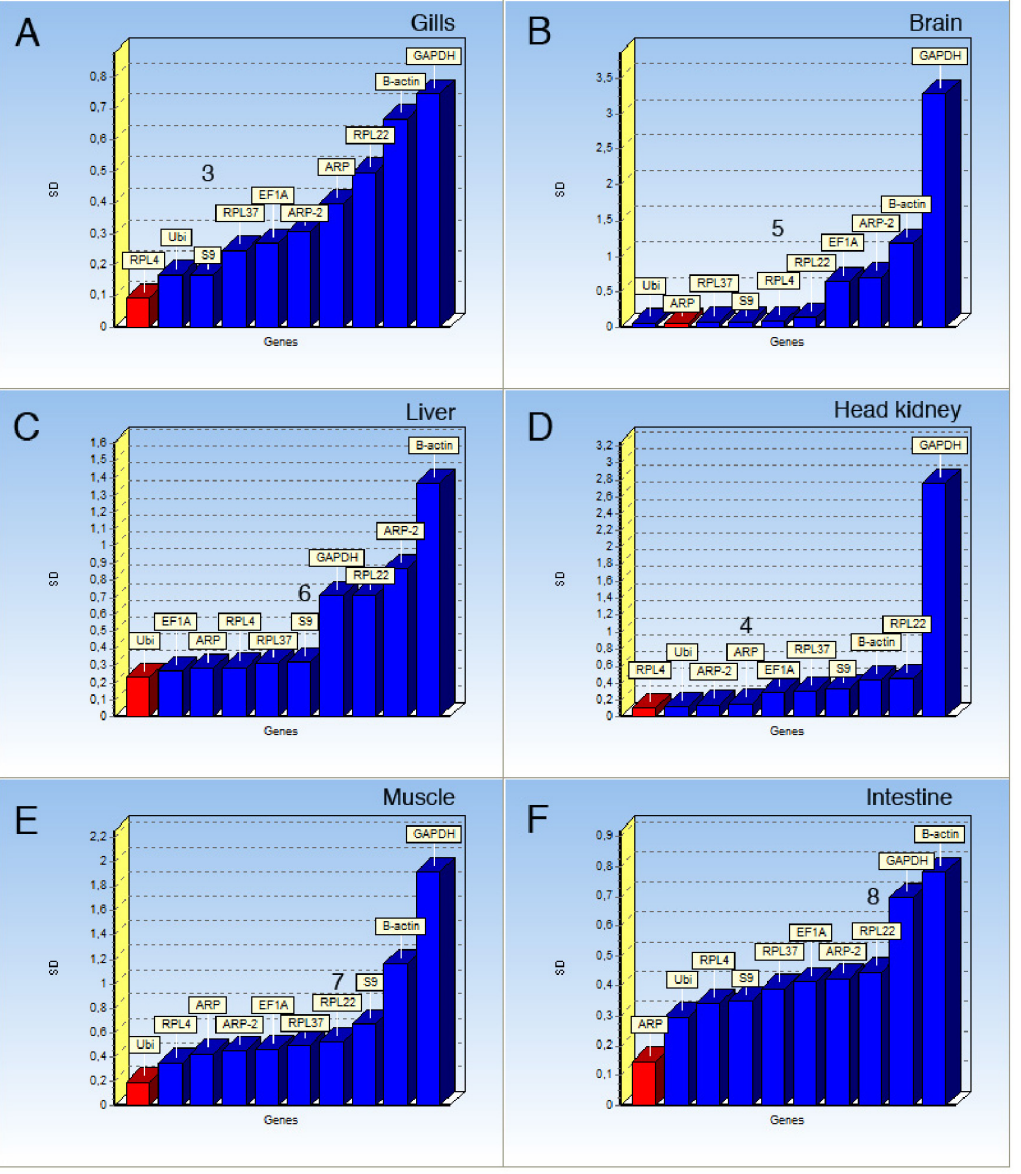
*NormFinder *ranking. A) Gills, B) Brain, C) Liver, D) Head kidney, E) Muscle and F) Intestine. The optimal number of genes suggested used for normalization by *NormFinder *is shown for each tissue.

Since *geNorm *uses a pair-wise comparison approach, co-regulated genes belonging to the same pathway or system with a similar expression profile tend to get too good score [6,11]. The *NormFinder *software, on the other hand, ranks the genes on a model-based approach. If the expression profiles suggest that several candidate genes are co-regulated, a model-based evaluation method should be considered. Analyzed with *NormFinder*, RPL4 was the most stable gene in gill tissue, followed by Ubi and S9 (Fig. 2A). RPL37, suggested as one of the top-ranking genes with *geNorm*, was ranked number 4 with *NormFinder*. Both *geNorm *and *NormFinder *rank GAPDH and ACTB as the least suitable reference genes in gills. In brain tissue, Ubi, ARP and RPL37 are ranked as the most suitable normalization genes, whereas GAPDH and ACTB again have the least stable expression profiles (Fig. 2B). S9, RPL4 and RPL22 are also considered to be suitable normalization genes in brain tissue according to the *NormFinder *tool. Ubi ranks best in liver tissue, followed by EF1A, ARP, RPL4, RPL37 and S9, all with almost the same expression profile stability (Fig. 2C). In liver the two structural proteins ARP-2 and ACTB were the least stable genes. RPL4, Ubi and ARP-2 were the most stable reference genes in head kidney tissue (Fig. 2D). The expression of GAPDH in head kidney varied a lot between the examined individuals, again putting a question mark on the use of this gene as a reference gene in qRT-PCR studies. In muscle tissue Ubi, RPL4 and ARP were the most stable reference genes according to *NormFinder*, with GAPDH and ACTB ranking worst (Fig. 2E). ARP, Ubi and RPL4 were the best reference genes in intestinal tissue evaluated using *NormFinder*, and ACTB and GAPDH ranking worst (Fig. 2F). The *NormFinder *software also suggest the optimal number of genes that should be included in order to calculate the normalization factor, to be used for normalization of target genes. These numbers are presented for each tissue in Fig. 2.

**Figure 3:**
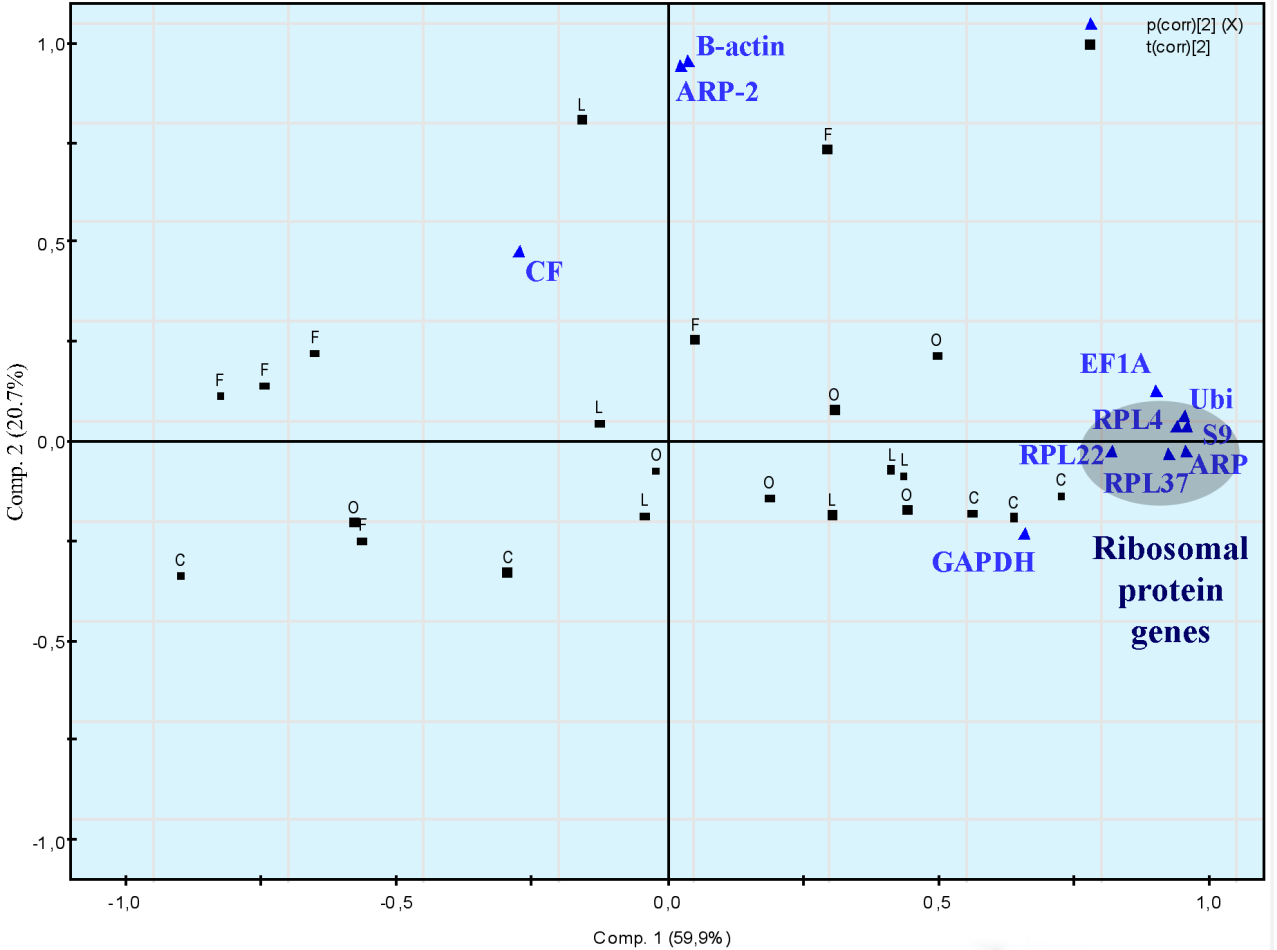
Population-specific principle component analysis (PCA) of gene expression in liver in order to check if fish from the contaminated sites group together. Ribosomal genes highlighted. Gene names, populations (C = Control, F = Farmed fish, L = Store Lungegårdsvann and O = Odda/Sørfjorden) and CF = Condition Factor are presented in the figure. PCA applied to the entire data set (component 1 and 2) model explaining r^2 ^= 0.81 and predicting Q^2 ^= 64% of the data variation. 95% confidence intervals. Centered data.

In all six examined tissues, both *geNorm *and *NormFinder *rank genes encoding ribosomal proteins and Ubi as the best candidate reference genes for normalization of qRT-PCR data. There are discrepancies between the two algorithms, but Ubi and ARP are among the best ranking genes analyzed with both methods in all tissues. In order to check if the selected genes encoding ribosomal proteins have equal expression profiles and are co-regulated, distribution patterns were investigated with principal component analysis (PCA). The *geNorm *algorithm is highly dependent on the assumption that none of the genes being analyzed are co-regulated [5]. Only in liver tissue did the genes encoding ribosomal proteins group tight together in the PCA plot (Fig. 3). In liver tissue Ubi, EF1A and GAPDH are grouping together with these genes, whereas the genes encoding the structural proteins ACTB and ARP-2 clearly deviates from the other genes.

Many fish studies has shown that GAPDH expression varies a lot between individuals and under various physiological conditions [12-15], so it was not surprising to find that this gene ranked among the least stable genes in all six examined tissues. Still, GAPDH should be considered included as a reference gene under certain experimental conditions or in specific tissues [16]. It was more surprising to find that ACTB also was ranked as one of the two least stable genes in all tissues. Genes encoding structural proteins like actins have in many ways replaced GAPDH as the golden standard in gene expression analysis [14]. Apparently, in Atlantic cod tissues ACTB should be used with caution as a reference gene. Especially in liver tissue, were the ACTB and ARP-2 genes encoding structural proteins were ranked as the two least stable genes.

In order to compare the *geNorm *and *NormFinder *results with an independent ranking method, the data were also analyzed with the *Bestkeeper *tool [7]. *Bestkeeper *uses a pair-wise correlation analysis of all pairs of candidate genes, and calculate the geometric mean of the best suited ones. The *Bestkeeper *ranking is shown in Table 3. Overall, the *Bestkeeper *results are in line with the *geNorm *and *NormFinder *data, with minor deviations. For example, in muscle tissue EF1A is ranked as the most stable gene. GAPDH and ACTB are again ranked as the least stable genes in all tissues. Analyzing reference genes in virus infected cells, Radonic et al. [17] concluded that the *Bestkeeper *tool gave results that slightly deviated from, but nevertheless corresponded to, those obtained using *geNorm*. Thus, under most experimental conditions it is appropriate to evaluate reference gene stability with only one of these tools.

**Table 3:** Evaluation of the usefulness of six potential reference genes in eight tissues of Atlantic cod ranked by the *Bestkeeper *software. 1 (bold) = best, 10 = worst. Six individuals from four populations (one with n = 5, total n = 23) were analyzed for 10 genes in six tissues. Ranking of reference genes according to the *Bestkeeper *software in six tissues of Atlantic cod. n = 23.

Gene	**Gills**	**Brain**	**Liver**	**Head kidney**	**Muscle**	**Intestine**
**ACTB**	10	9	10	9	9	10
**EF1A**	8	8	6	8	**1**	9
**ARP-2**	7	6	5	6	2	5
**RPS9**	4	3	4	3	7	6
**ARP**	**1**	**1**	2	4	5	3
**GAPDH**	9	10	9	10	10	8
**Ubi**	3	2	**1**	2	3	2
**RPL4**	6	5	8	7	8	7
**RPL22**	5	7	3	5	4	4
**RPL37**	2	4	7	**1**	6	**1**

**Figure 4:**
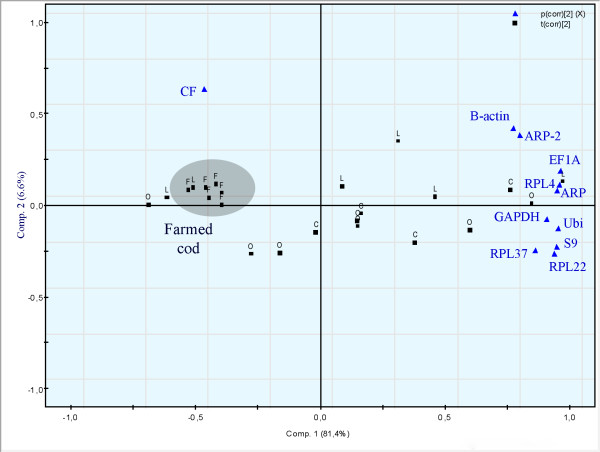
Population-specific principle component analysis (PCA) of gene expression in intestinal tissue in order to check if fish from the contaminated sites group together. Farmed cod highlighted. Gene names, populations (C = Control, F = Farmed fish, L = Store Lungegårdsvann and O = Odda/Sørfjorden) and CF = Condition Factor are presented in the figure. PCA applied to the entire data set (component 1 and 2) model explaining r^2 ^= 0.88 and predicting Q^2 ^= 74% of the data variation. 95% confidence intervals. Centered data.

**Figure 5:**
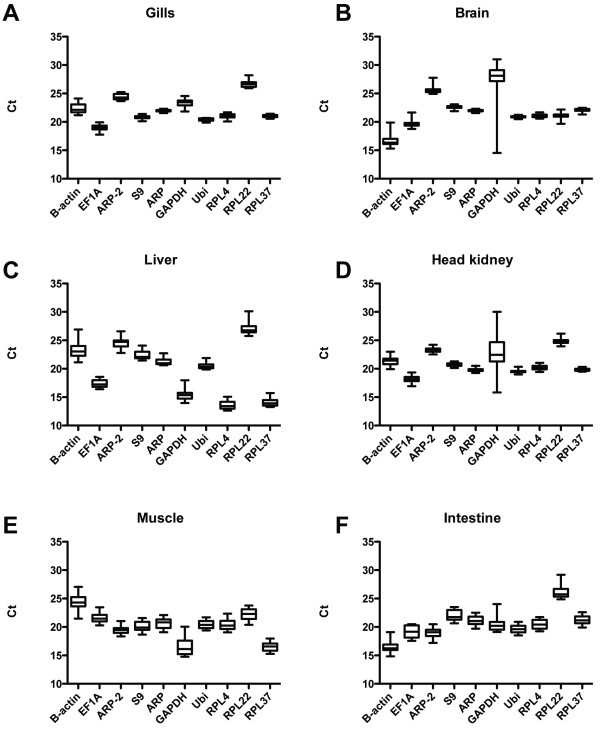
Tissue distribution of the studied genes as indicated by the raw Ct values. A) Gills, B) Brain, C) Liver, D) Head kidney, E) Muscle and F) Intestine.

One of the aims of this investigation was to evaluate if potential reference genes are stable in a certain tissues across different populations inhabiting contaminated areas. The expression of candidate genes might be differentially regulated in fish living in strongly contaminated location, for example under conditions were they are experiencing physiological stress that might affect the metabolism. Using PCA, Atlantic cod from the two contaminated locations did not group together (data not shown). Instead, in intestinal tissue individuals from the farmed population grouped together (Fig. 4). Farmed fish are fed on a daily basis with a standard diet, so it is not surprising that the expression of the selected 10 genes group together in a PCA plot in intestinal tissue. Our results therefore suggest that the studied candidate reference genes can be used for normalization of qRT-PCR data across different wild populations of Atlantic cod. Our data, based on *geNorm *and *NormFinder *calculations, suggest that the Atlantic cod Ubi and ARP genes that have been tested in the present study may be good candidate reference genes in brain, liver, muscle and intestinal tissues. The study also suggest that ACTB gene should be applied with caution in qRT-PCR examinations of Atlantic cod, as this gene was ranked as on of the least stable in all examined tissues, and also showed considerable variation from sample to sample (Fig. 5).

In conclusion, this study suggests that Ubi and ARP are potential candidate reference genes in qRT-PCR examinations of relative gene expression in gill, brain, liver, head kidney, muscle and intestine tissues of wild populations of Atlantic cod.

## Authors' contributions

PAO was responsible for the experiment, data analysis and wrote the manuscript. KKL participated in planning, sampling, and contributed throughout the experimental process. LS contributed in the statistical analysis of the data. All authors read and approved the final manuscript.
