# Correction: Selection of reference genes for qRT-PCR examination of wild populations of Atlantic cod Gadus morhua

**DOI:** 10.1186/1756-0500-4-456

**Published:** 2011-10-27

**Authors:** Pal A Olsvik, Liv Softeland, Kai K Lie

**Affiliations:** 1 National Institute of Nutrition and Seafood Research, Nordnesboder 2, N-5005 Bergen, Norway

## 

Following the publication of our article[1], an error in Table 2 was noted, the wrong accession number and primer sequences were stated for the EF1A assay.

The GenBank accession number was incorrectly stated as:

EX721840

Forward primer: CCCTGTGGAAGTGGCTGAAG

Reverse primer: CATCCAAGGGTCCGTATCTCTT

The correct GenBank accession number should be:

EX722124 Forward primer: CGGTATCCTCAAGCCCAACA Reverse primer: GTCAGAGACTCGTGGTGCATCT

We apologise for any inconvenience this may have caused.
